# Fatal bilateral pneumothoraces complicating dyskeratosis congenita: a case report

**DOI:** 10.1186/1752-1947-3-6622

**Published:** 2009-03-26

**Authors:** Adel Boueiz, Marwan S Abougergi, Carlos Noujeim, Edmond Bou Assaf, Ghassan Jamaleddine

**Affiliations:** 1 Division of Pulmonary & Critical Care Medicine, Department of Medicine, Johns Hopkins University School of Medicine, Baltimore, MD 21224, USA; 2 Department of Medicine, Johns Hopkins University School of Medicine, Baltimore, MD 21224, USA; 3 Division of Pulmonary & Critical Care Medicine, Department of Medicine, American University of Beirut, Beirut, Lebanon; 4 Department of Medicine, State University of New York, Downstate, Brooklyn, NY, USA; 5 Division of Pulmonary & Critical Care Medicine, Department of Medicine, State University of New York, Downstate, Brooklyn, NY, USA

## Abstract

**Introduction:**

Dyskeratosis congenita is a rare genodermatosis, characterized by a triad of reticular skin pigmentation, nail dystrophy and leukoplakia of mucous membranes. It is also associated with a variety of non-cutaneous abnormalities such as bone marrow failure, malignancy and pulmonary complications. Among its wide range of clinical manifestations, fatal pneumothorax has rarely been reported.

**Case presentation:**

We report the case of a 31-year-old Lebanese woman with dyskeratosis congenita who succumbed to devastating bilateral pneumothoraces.

**Conclusion:**

Careful surveillance of patients with dyskeratosis congenita is required as incipient respiratory failure due to pneumothorax may be successfully treated if detected at an early stage.

## Introduction

Dyskeratosis congenita (DC) is an inherited syndrome that was first described by Zinsser in 1910, and further characterized by Engman (1926) and Cole et al. in 1930 [1]. It is estimated to occur in 1 in 1 million people, with death occurring at a median age of 16 [1]. It typically presents with a triad of mucocutaneous abnormalities consisting of reticulated skin hyperpigmentation, nail dystrophy (both occurring in almost 100% of cases), and mucosal leukoplakia (80% of cases) [1]. Other features occur with lower frequencies and involve virtually every organ system (Table 1) [1]-[3]. Although rare (approximately one case per million individuals) [4], DC is a serious and usually fatal condition. The main causes of death are bone marrow failure/immunodeficiency (~60-70%), pulmonary complications (~10-15%), and malignancy (~5-10%) [2].

**Table 1:** Clinical abnormalities reported, so far, in association with dyskeratosis congenita

Organ system	Defect or abnormality
Skin	Abnormal skin pigmentation
	Chronic dermal ulcer
	Hyperhidrosis
Nail	Nail dystrophy
Oral cavity	Extensive dental caries
	Dental loss
	Leukoplakia
	Squamous carcinoma of the mouth
Hair	Premature hair
	Alopecia
	Grey hair
	Sparse eyelashes
Lungs	Asthma
	Pulmonary fibrosis
	Hepatopulmonary syndrome
	Pulmonary microvascular abnormalities
	Bronchiectasis
	Fibrocystic dysplasia
	*Pneumocystis jiroveci* pneumonia
	Chronic pneumonitis
Gastrointestinal	Esophageal stricture
	Peptic ulceration
	Enteropathy
	Liver disease (cirrhosis, portal hypertension)
	Gastrointestinal adenocarcinoma
Genitourinary	Hypogonadism
	Undescended testes
	Urethral stricture
	Phimosis
Central Nervous System	Learning difficulties
	Developmental delay
	Mental retardation
	Ataxia
	Cerebellar hypoplasia
	Cerebellar malformation
	Microcephaly
	Peripheral neuropathy
Hematologic	Bone marrow failure
	Hodgkin's lymphoma
Skeletal	Osteoporosis
	Aseptic necrosis
	Scoliosis
	Short stature
Ophthalmic	Epiphora
	Chronic keratoconjunctivitis
Gynecologic and Obstetric	Intrauterine growth retardation
	Vaginal squamous cell carcinoma
	Cervical squamous cell carcinoma
ENT	Deafness
	Laryngeal carcinoma
	Nasopharyngeal carcinoma

Pulmonary involvement occurs in as many as 20% of DC patients [5]. A wide spectrum of respiratory manifestations has been reported, including asthma, pulmonary fibrosis, hepatopulmonary syndrome, pulmonary microvasculature abnormalities, bronchiectasis, fibrocystic dysplasia, *Pneumocystis jiroveci* pneumonia and chronic pneumonitis [6]-[9]. In this article, we describe an unusual case of fatal bilateral spontaneous pneumothoraces in a woman with DC.

## Case presentation

A 31-year-old Lebanese woman presented to our medical centre with acute onset of shortness of breath and pleuritic chest pain. Her medical history dated back to 20 years before presentation, when she started developing neck and upper chest skin hyperpigmentation along with progressive hair loss. A few years later, she was found to have pancytopenia and bone marrow hypoplasia and was diagnosed with DC. Since that time, she had been receiving blood transfusions regularly in another medical institution. Her family history was remarkable for parental consanguinity and a diagnosis of DC in one sister who died of an unknown DC-associated complication. The review of systems was non-contributory.

Physical examination showed a tall, slender woman with diffuse alopecia, loss of eyebrows and areas of reticulate hyperpigmentation over the neck and upper chest. Patches of leukoplakia were present on the tongue with atrophy of the glossal papillae. She was dyspneic at rest but hemodynamically stable. Hyper-resonance, decreased breath sounds over the left lung and *velcro* crackles were noted on lung examination. In addition, there was atrophy and longitudinal ridging of the nail beds. No digital clubbing was noted. Cardiovascular, abdominal, and neurological examinations were normal.

**Figure 1:**
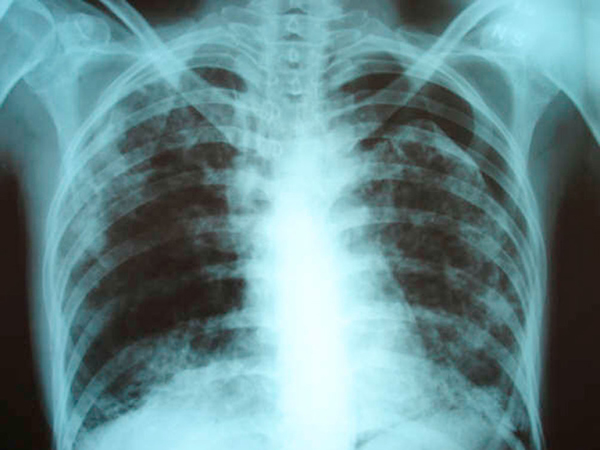
**Chest X-ray film taken on the first admission to our institution showing large left pneumothorax estimated at 35% and reticulonodular infiltrates involving both lung fields**.

**Figure 2:**
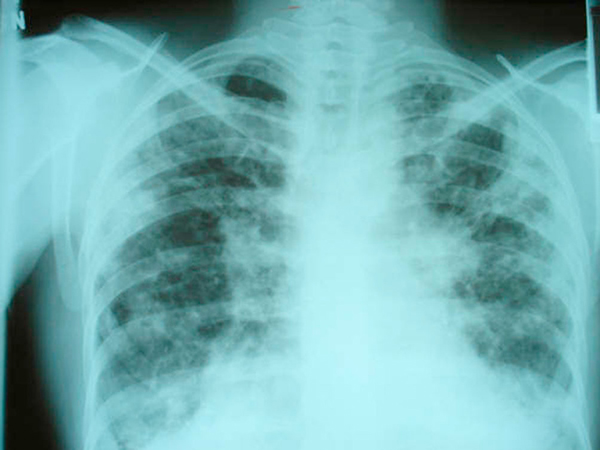
**Chest X-ray film taken 1 year before the patient's first admission to our institution, showing patchy areas of increased density in the periphery of both lung fields, hyperinflation of the lungs and areas of honeycombing in the right lower lobe consistent with interstitial fibrosis**.

Chest X-ray revealed a large left pneumothorax estimated at 35% and reticulonodular infiltrates involving both lung fields (Figure 1). An X-ray done 1 year earlier showed bilateral peripheral patchy hyperdensities associated with pulmonary hyperinflation and areas of honeycombing in the right lower lobe consistent with interstitial fibrosis (Figure 2).

A chest tube was inserted into the left pleural space and placed under negative pressure. Five days later, the left lung had not expanded despite the absence of a bronchopleural leak. The patient continued to be hemodynamically stable. Her shortness of breath and pain improved slightly. The decision was then made to remove the chest tube and discharge the patient home.

**Figure 3:**
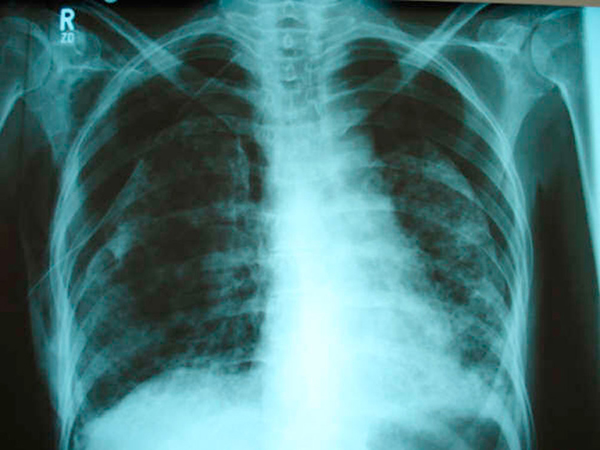
**Chest X-ray film taken 3 months after the patient's first admission to our institution, showing a right-sided pneumothorax, estimated at 50%, with persistence of the left pneumothorax; right chest tube in place**.

Three months later, she presented again with sudden onset of chest discomfort. An X-ray of the chest at that time showed a right-sided pneumothorax estimated at 50%, with persistence of the old left pneumothorax. A chest tube was inserted in the right pleural space (Figure 3). She was later intubated and placed on Assist-Control low-tidal volume mechanical ventilation for worsening hypoxic respiratory failure. Her initial FiO_2_ requirement was 80% for 3 days, and then progressively decreased to 60%. The positive end-expiratory pressure (PEEP) was maintained at 5mmHg. Over a 3-week period, although the chest tubes were in place and no bronchopleural air leakage was noted, there was no significant improvement in either of the bilateral pneumothoraces. The PaO_2_/FiO_2_ ratio was constantly rising, reaching around 360 a month into the ICU admission, when the patient passed away due to intractable hypoxic respiratory failure. The poor lung re-expansion was documented bilaterally by serial X-rays, and there was no evidence of ventilator-associated pneumonia or any additional comorbidity.

## Discussion

DC is a multisystem genodermatosis where distinctive mucocutaneous lesions occur in combination with bone marrow failure, pulmonary disease and an increased risk of malignancy [1]. The mode of inheritance is an X-linked recessive pattern in the majority of cases [10]. DC continues to stimulate clinical and research interest because, although rare, it is devastating for the patient and his/her family [11].

Our patient presented with several typical features of DC: reticulate pigmentation of the neck and upper chest with areas of spared skin (confetti spots), dystrophic nails, diffuse alopecia, together with the characteristic bone marrow failure and interstitial fibrosis. The course of her disease was remarkable for the development of spontaneous bilateral pneumothoraces leading to intractable hypoxic respiratory failure and death despite chest tube insertion, application of negative pressure and use of mechanical ventilation. The sister's DC diagnosis and the parental consanguinity suggest an autosomal recessive rather than the more usual X-linked inheritance mode [10]. Subsequently, three other members of the same family were diagnosed with DC, making autosomal recessive inheritance even more likely. Unfortunately, genetic testing was not available at that time to confirm the specific genotype or inheritance mode.

Pulmonary disease in patients with DC has rarely been reported and poorly documented [7]. However, pulmonary involvement may be underestimated because many DC patients die at an early age, before the onset of respiratory symptoms. In addition, DC patients may present with primary respiratory complaints and the condition goes unrecognized because of minor dermatologic manifestations [10].

A 1973 review of the literature identified fibrotic lung changes in only 2 of 35 reported patients [12]. The DC registry established at the Hammersmith Hospital (London) reported in year 2000 that 20.3% of 148 patients from 92 families exhibited reduced diffusion capacity and/or restrictive defects on pulmonary function testing [2]. So far, a total of eight patients have been reported with biopsy-proven pulmonary fibrosis [13]. Other reported pulmonary manifestations of DC include asthma, hepatopulmonary syndrome, pulmonary microvasculature abnormalities, bronchiectasis, fibrocystic dysplasia, *Pneumocystis jiroveci* pneumonia and chronic pneumonitis [6]-[9].

Secondary spontaneous pneumothorax is a condition mainly seen in patients with asthma, chronic obstructive pulmonary disease (COPD), infections (including abscess, tuberculosis, *Pneumocystis jiroveci* pneumonia) and malignancies. It is less commonly reported in patients with interstitial lung disease as part of specific syndromes such as lymphangioleiomyomatosis and histiocytosis X. It has rarely been reported in patients with DC. To the best of our knowledge, the only report of a pneumothorax complicating DC was that by Verra *et al.*, in 1992 [7]. The patient in question was admitted for acute respiratory failure precipitated by a unilateral, left-sided pneumothorax and passed away 10 days later despite chest tube insertion and oxygen therapy. Similarly, our patient developed fatal bilateral pneumothoraces refractory to chest tube insertion, application of negative pressure and use of mechanical ventilation. In both cases, a pneumothorax occurring in the setting of DC was fatal.

The pathophysiology of the different DC manifestations remains incompletely characterized despite significant advances in our understanding of the genetics and biology of this disease during the last decade [1]. It is currently believed that DC arises principally from defective telomere maintenance, resulting in chromosomal shortening and gene loss during cell replication ultimately leading to cell apoptosis, particularly in highly proliferative tissues such as the hematologic and dermatologic systems [11]. Utz *et al.* speculated that pulmonary fibrosis in this setting is secondary to an abnormal fibroblast function [13]. Once established, this pulmonary fibrosis may lead to alveolar wall degeneration, cyst formation and rupture, and therefore to a pneumothorax. Furthermore, since DC has been shown to be associated with pulmonary microvascular abnormalities [7], it is also possible that pneumothoraces are the result of pulmonary infarctions. The absence of lung re-expansion after chest tube insertion observed in the course of our patient's illness can be multifactorial: for example, extensive fibrosis could have permanently deformed the lung and prevented significant re-expansion, or infarction involving a significant part of the lung parenchyma could have delayed healing.

Traditionally, treatment options for bone marrow failure in patients with DC include anabolic steroids (for example, oxymetholone), granulocyte macrophage colony-stimulating factor, granulocyte colony-stimulating factor and erythropoietin [14]. However, this kind of therapy has been associated with only a transient increase in blood counts and serious adverse effects [15]. Hematopoietic stem cell transplantation has a limited success rate because of a high prevalence of fatal pulmonary complications, mainly in patients with significant pre-existing pulmonary involvement. No data are available, thus far, regarding the management of pulmonary manifestations in DC patients. The emerging insights into the molecular biology of this disease will hopefully provide new opportunities for preventive and therapeutic interventions.

## Conclusion

In summary, we describe the case of a patient with typical DC symptoms who presented with bilateral pneumothoraces, refractory to existing medical treatment. This is the second reported pneumothorax occurrence in the context of DC. In both reports, the outcome was fatal despite maximal medical therapy. Therefore, a high degree of suspicion of this and other potential pulmonary complications in patients with DC, and surveillance pulmonary function testing and chest imaging are warranted for early effective interventional strategies.

## Consent

Written informed consent was obtained from the patient's next-of-kin for publication of this case report and any accompanying images. A copy of the written consent is available for review by the Editor-in-Chief of this journal.

## Competing interests

The authors declare that they have no competing interests.

## Authors' contributions

AB and MSA contributed to interpretation of data, literature search and drafting of the manuscript. CN contributed to data collection and literature search for the manuscript. EBA contributed to patient care, drafting the manuscript and literature review. GJ contributed to patient care, revision and final approval of the manuscript. All authors read and approved the final manuscript.
